# Fifty Years of Killing and Letting Die: On the Limits of Philosophical Bioethics

**DOI:** 10.1111/bioe.13351

**Published:** 2024-09-24

**Authors:** Joona Räsänen, Matti Häyry

**Affiliations:** ^1^ Department of Philosophy, Contemporary History and Political Science, and Turku Institute for Advanced Studies University of Turku Turku Finland; ^2^ Department of Management Studies Aalto University School of Business Espoo Finland

**Keywords:** applied ethics, assisted dying, euthanasia, killing, letting die

## Abstract

In 1975, *The New England Journal of Medicine* published James Rachels' article ‘Active and Passive Euthanasia’. The argumentative method that Rachels introduced, the Bare Difference Argument (also known as the Contrast Strategy), became one of the most widely used tools in ethical reasoning. The argument, however, fails to show active euthanasia being morally permissible. It fails because Rachels takes the intuitions from the case where letting die is morally impermissible and applies the intuitions to cases where letting die is morally permissible. While it is possible to create thought‐experiments that are more analogous to euthanasia, in this respect, than Rachels' cases, they too are disanalogous to euthanasia with some of the relevant features. Creating the perfect analogy, however, would be a mistake too. Such a case would be too analogous; people would simply be divided on what kind of moral intuitions they would have. The problem thus highlights a methodological limit in philosophical bioethics and raises questions related to the roles of philosophical ethicists in the context of assisted dying.

Fifty years ago, *The New England Journal of Medicine* published James Rachels' (1975) article ‘Active and Passive Euthanasia’.^1^ The essay soon become a classic in the fields of medical ethics, bioethics, and applied philosophy, and the distinction between killing and letting die has been studied by philosophers and bioethicists ever since.^2^ The argumentative method that Rachels introduced became one of the most widely used tools in ethical reasoning.^3^ Rachels' essay had an impact on the debate on legalizing active euthanasia also, although the different responses to voluntary active medical euthanasia in different countries are arguably ideological.^4^

While Rachels' method can be used in many contexts,^5^ the argument does not seem to work in the context of euthanasia. While it might perhaps successfully show something, the argument does not show that active euthanasia is morally permissible. We will explain.

Rachels brought forth what has now become known as the Bare Difference Argument (also known as the Contrast Strategy^6^). The form of this argument, which has a remarkable resemblance to the argumentative method in sciences,^7^ involves considering two imaginary cases in which there are no differences present—except the one in which moral relevance we are interested in. For Rachels, the difference was the distinction between killing and letting die.

The argumentative method says that, when considering the pair of cases, if the difference makes no moral difference in our ethical evaluation, then it cannot be that the difference is *itself* a morally important matter. In the cases where the only difference is whether the person kills or lets die, if we do not judge the killer doing something worse than the person who merely lets die, then the difference between killing and letting die is itself a morally irrelevant matter.

To illustrate Rachels' argument, consider the following, slightly adapted cases from Rachels. When you do, imagine that you know all of the following and nothing more—of any relevance to your ethical evaluation.

*Killing*. Smith will gain a large inheritance if his six‐year‐old cousin dies. One evening, while the child is taking a bath, Smith sneaks into the bathroom, drowns the child, and makes it look like an accident.

*Letting Die*. Jones will gain a large inheritance if his six‐year‐old cousin dies. One evening, while the child is taking a bath, Jones sneaks into the bathroom planning to drown the child. But, as Jones enters the bathroom, the child hits his head and falls face down into the water. Jones stands ready to kill the child if necessary. But the child dies on his own.

After presenting the above cases, Rachels then asked did either man behave better from a moral point of view? Smith killed the child, whereas Jones “merely” let the child die. That is the only difference between the cases. For instance, the people's motives were the same and the result of their actions/inactions was similar. So, did one of them act morally worse than the other? Rachels' answer is no.

If this ethical intuition is widely shared (as Rachels assumed it is), then, killing is not worse than letting die. If—and, according to Rachels, since—that is the case, then active euthanasia is no worse than passive euthanasia. This is because the only difference between active euthanasia and passive euthanasia is precisely the difference between killing and letting die—a morally irrelevant distinction.

However, the neglected problem with the argument is that Rachels takes the intuitions from the case where letting die is clearly morally *impermissible*—the case where Smith lets his cousin die in the bath—and uses the moral intuitions generated by this case as evidence for cases where letting die is morally *permissible*, such as passive euthanasia. We cannot reason from the conclusion that killing is no worse (than letting die) when it is *impermissible* to let die, to another conclusion that states that killing is no worse (than letting die) when it is *permissible* to let die.

To highlight the significance of this problem, we want to point out that Rachels is not the only one who made the same mistake. Michael Tooley argued that killing is no worse than letting die by presenting cases of two sons who are looking forward to the death of their wealthy father and decide independently to poison him.^8^ One son puts poison in his father's whiskey, and is discovered doing so by the other, who was just about to do the same. The latter son then allows his father to drink the poisoned whiskey and refrains from giving him the antidote, which he happens to possess—thus letting him die. Tooley concludes that the brothers acted equally wrong—a conclusion that is easy to agree on. However, again, the problem is that it is obviously immoral to kill in the case. It is wrong to poison one's father—thus, Tooley's poison cases tell us very little, if anything, on how we should think of the permissibility of active euthanasia since passive euthanasia is, according to many, permissible. Tooley, like Rachels, only showed that killing is no worse than letting die—when letting die is impermissible.

So, Rachels' argument was as follows:

P1: Passive euthanasia is morally permissible.

P2: Other things being equal, killing is no worse than letting die.

P3: The only difference between active euthanasia and passive euthanasia is that the former is an instance of killing and the latter is an instance of letting die.

C: Active euthanasia is morally permissible.

However, as seen, Rachels' Bare‐Difference Argument, which aims to support P2, supports a different premise instead. That premise is the following.

P2_B_: Other things being equal, killing is no worse than letting die, in cases where letting die is impermissible.

Because letting die is permissible (not impermissible) in passive euthanasia—as stated in P1, the conclusion (C) does not follow. To understand why, consider the following argument.

P1: Not telling the truth is morally permissible.

P2: Other things being equal, lying is no worse than not telling the truth.

P3: The only difference between lying and not telling the truth is that the former is an instance of speaking against the truth and the latter is not.

C: Lying is morally permissible.

P1 is a plausible assumption,^9^ and P2 can be supported by the following cases.

*Lying*. Anna's six‐year‐old son wants to know if his drawing of a polar bear looks good. Anna thinks no; the drawing looks like a three‐legged dog. But she does not want to make him feel bad. So, she says that the drawing looks good. Her son is happy because of that.

*Not Telling the Truth*. Bella's six‐year‐old son wants to know if his drawing of a polar bear looks good. Bella thinks no; the drawing looks like a three‐legged dog. But she does not want to make him feel bad. So, she remains silent. Her son is happy because of that.

Following Rachels, we can now ask did either woman behave better from a moral point of view? Anna lied to her son, whereas Bella “merely” did not tell the truth. That is the only difference between the cases. For instance, the people's motives were the same and the result of their actions/inactions was similar. So, did one of them act morally worse than the other? We think not. And we assume that, surely, most people agree that Anna behaves no worse in the examples than does Bella even though Anna lied while Bella did not speak the truth.

Now we could claim to have reached the conclusion: Lying is morally permissible. Our reasoning for reaching the conclusion is that we did not judge what Anna did, to be morally worse than what Bella did, and the only difference between the cases was that Anna lied while Bella did not speak the truth since she did not speak at all.

But now consider the following cases.

*Silent Doctor*. Clara is a medical doctor whose patient asks for advice on whether he should drink colloidal silver water. The patient suspects it might be healthy, but Clara knows better; it is not. In fact, it could be dangerous. The patient will drink the colloidal silver water unless Clara tells her not to. Clara does not say anything, so the patient drinks colloidal silver water.

*Lying Doctor*. Dana is a medical doctor whose patient asks for advice on whether he should drink colloidal silver water. The patient suspects it might be healthy, but Dana knows better; it is not. In fact, it could be dangerous. The patient will drink the colloidal silver water unless Dana tells her not to. Dana tells her patient it is perfectly safe and healthy to drink the colloidal silver water, so the patient drinks it.

Here, it seems that what Dana did is morally worse than what Clara did—even though the distinction between lying and remaining silent is the bare difference between the two cases.

So why do we now think differently than we did when we compared our intuitive reactions between *Lying* and *Not Telling the Truth*? Well, in the latter cases, it was impermissible to remain silent, while in the former cases, it was permissible to do so.

So, we cannot claim that lying is always morally equal to being silent—even though it sometimes is. Likewise, we cannot claim that killing is always morally equal to let die—even though it sometimes is. Whether lying makes mere obmutescence morally worse depends on whether it is morally permissible to be silent in the first place. It is a plausible claim that whether killing makes ending a life worse depends on whether it is morally permissible to let die in the first place too.

Back to Rachels. What Rachels' argument perhaps shows is that killing is no worse than letting die when letting die is impermissible. Consider, for instance, a case where a madman kidnaps a child and lets her starve to death. It seems that killing the child instead of letting her starve to death is not morally worse—in fact, perhaps it is morally better to kill the child because sometimes, it would be better to end the life actively to avoid any further suffering.^10^ So it seems that the cases of the two cousins support this: ‘Other things being equal, killing is no worse than letting die—if letting die is *impermissible*.’

Be that as it may, since Rachels' aim was to argue that active euthanasia is morally permissible and should, therefore, be legalized,^11^ he should have created cases that would support the following claim: ‘Other things being equal, killing is no worse than letting die—if letting die is *permissible*’.

Fiona Woollard presents such cases. Consider the following thought‐experiments.^12^

*Rolling Boulder*. You are a mountain rescue worker in the process of taking two severely injured men, Alistair and Bryan, to hospital. On the way to the hospital, you notice a third man, Charlie, trapped in the path of a large boulder that is rolling down the hill. If you stop to help Charlie, then your arrival at the hospital will be delayed and Alistair and Bryan will die. If you do not stop to help, Alistair and Bryan will be saved but Charlie will be hit by the boulder and crushed to death.

*Pushing Boulder*. Same as before, but now the boulder is blocking your route to the hospital and the only way you could get through in time is to push the boulder towards Charlie. Detouring round the boulder or stopping to free Charlie would delay you too long. Your only options are to push the boulder towards Charlie – in which case Charlie will be crushed by the boulder but you will be able to save Alistair and Bryan—or to abandon the rescue attempt—in which case Charlie will eventually be rescued but Alistair and Bryan will die.

Woollard rightly notes that there is a moral difference between the two cases. While it seems permissible to refuse to stop and help Charlie so that you can save Alistair and Bryan, it does not seem permissible to push the boulder towards Charlie to save Alistair and Bryan. Thus, this analysis seems to show that killing is worse than letting die—when letting die is permissible.

However, the problem with Woollard's cases is that while they are analogous with active euthanasia with one morally relevant feature (in both, letting die is permissible), they are disanalogous with other relevant features. For instance, in active euthanasia, we are not saving multiple people by killing one; we are ending the life of one patient to reduce their suffering and respect their autonomous choice to decide about their own life. Because of such disanalogies, it seems that the intuitions from Woollard's cases cannot be used as guidance for the euthanasia debate any more than the intuitions from Rachels' cases of two cousins.

To bring home the relevance of the moral (im)permissibility of the choice, considering another distinction, ignored by Rachels, could be useful. Forms of euthanasia conceptually include, in addition to active and passive, also direct and indirect. In the direct form, the physician or medical team typically administers a lethal drug that kills the patient. In the indirect form, the physician or medical team increases pain medication with the intent of alleviating pain and anguish but with the foreseen consequence of hastening the patient's death. Employing the doctrine of double effect,^13^ some have argued that ending the pain by ending the life intentionally is impermissible, whereas ending the life unintentionally (this is a debated point that does not concern us here) is permissible.

In terms of our criticism of Rachels' argument, the focal concept is intentionality. All the cousins, mothers, and medical doctors of our example pairs intend—want, wish, are alright with—the death or being‐lied‐to of those affected by their actions. Whether doing or omitting, killing or letting die, or be uninformed, they accept the bad consequence of their choice. If that is what makes decisions impermissible, Rachels' original bare difference remains intact but testifies for the wrongness of euthanasia, also what he calls passive euthanasia. To see that, we only need to start the analysis from the other end.

These points raise a broader worry about the role of philosophy in medical ethics. As the discussion of the permissibility of assisted dying continues, the perfect analogy with euthanasia is yet to be found. And if the perfect analogy is found, it might be *too* analogous, thus telling nothing new about our moral intuitions; people would simply have different intuitive reactions to the analogy, in the same way as people have different intuitions to active euthanasia. Consider, for instance, someone revising Rachels' cousin cases or Woollard's boulder cases to the extent that every detail of them would eventually match with the cases of active and passive euthanasia. Our intuitions would then be useless because those cases would *be* the cases of active and passive euthanasia; moral intuitions generated by such cases are not an explanation of anything since they would be in need of an explanation.

In view of the goals that ethicists should pursue in the context of assisted dying, the failure of Rachels’ Bare Difference Argument shows that at least philosophical bioethicists should not try to pontificate on clinical cases based on made‐up conceptual differences. The arguments by analogy used by ethicists should be more sensitive to context and should not neglect the pragmatic dimensions of the practices in which the individual actions, on which the analogical arguments draw, are embedded. Philosophical bioethicists should, rather, aim at having a nuanced view of real‐life situations like assisted‐dying choices and their ethically relevant dimensions *and* at making these dimensions known to decision‐makers on all levels—bedside, ward, hospital, health care provision, and legislation. The conclusion of such analyses can well be that matters remain contested^14^—but if that is the reality, then bioethicists would overstep their professional roles by claiming otherwise.^15^

What philosophical ethicists can and perhaps should do, however, is to make sure that the arguments presented are logically consistent and conceptually coherent—especially regarding agent's other commitments and beliefs.^16^ Ethicists with background in philosophy are very much needed for their critical outlook dissecting and systemizing skills that can help to clarify the discussions.^17^ But when it comes to normative guidance, it is false to assert that philosophical ethicists know better than others what to do.

## Endnotes

1 Rachels, J. (1975). Active and passive euthanasia. *The New England Journal of Medicine, 292*, 9(28), 78–80.

2 Thomson, J. (1975). Killing, letting die, and the “trolley problem”. *The Monist, 59*(2), 204–217; Nesbitt, W. (1995). Is killing no worse than letting die? *Journal of Applied Philosophy, 12*(1), 101–106; Asscher, J. (2008). The moral distinction between killing and letting die in medical cases. *Bioethics, 22*(5), 278–285; Hill, S. (2018). Murdering an accident victim: A new objection to the bare‐difference argument. *Australasian Journal of Philosophy, 96*(4), 767–778.

3 Kopeikin, Z. A. (2020). Bare‐difference methodology and a problematic separability principle*. Journal of Value Inquiry, 54*, 553–570.

4 Häyry, H. (1997). Bioethics and political ideology: The case of active voluntary euthanasia. *Bioethics, 11*(3‐4), 271–276.

5 Räsänen, J. (2021). Age and ageing: What do they mean? *Ratio, 34*(1), 33–43.

6 Kagan, S. (1998). The additive fallacy. *Ethics, 99*(1), 5–31.

7 Kopeikin, Z. A. (2021). Bare‐difference methodology and the scientific analogy. *Ratio, 34*(3), 171–182.

8 Tooley, M. (1980). An irrelevant consideration: killing and letting die. In B. Steinbock (Ed.), *Killing and letting die* (pp. 56–62). Fordham University Press.

9 This can be, of course, contested, but whether the premise is eventually true is irrelevant for making our point.

10 Kuhse, H. (1998). Critical notice: Why killing is not always worse–and is sometimes better–than letting die. *Cambridge Quarterly of Healthcare Ethics, 7*(4), 371–374.

11 While Rachels does not explicitly say that active euthanasia should be legalized, his statement that doctors should not give added authority and weight to the legal differentiation by ‘writing it into official statements of medical ethics’ could be interpreted as a plea to legalize active euthanasia. This interpretation arises from the widely agreed‐upon notion that official statements of medical ethics should respect what is legal and illegal in the particular community. We thank an anonymous reviewer at *Bioethics* for this remark.

12 Adapted from Woollard, F. (2012). The doctrine of doing and allowing I: Analysis of the doing⁄allowing distinction. *Philosophy Compass, 7*(7), 448–458.

13 McIntyre, Alison, “Doctrine of Double Effect”, The Stanford Encyclopedia of Philosophy (Winter 2023 Edition), Edward N. Zalta & Uri Nodelman (eds.), <https://plato.stanford.edu/archives/win2023/entries/double-effect/>

14 Häyry, M. (2018). Fear of life, fear of death, and fear of causing death: How legislative changes on assisted dying are doomed to fail. *Cambridge Quarterly of Healthcare Ethics*, *27*(1), 145–153.

15 Häyry, M. (2010). Rationality and the genetic challenge making people better? Cambridge University Press; Häyry, H., & Häyry, M. (1994). The nature and role of professional codes in modern society. In R.F. Chadwick, *Ethics and the Professions* (pp. 136–144). Aldershot, Brookfield, Hong Kong, Singapore and Sydney: Avebury.

16 Häyry, M. (2015). What exactly did you claim? *Cambridge Quarterly of Healthcare Ethics*, *24*(1), 107–112; Häyry, M. (2015). What do you think of philosophical bioethics? *Cambridge Quarterly of Healthcare Ethics*, *24*(2), 139–148.

17 Räsänen, J., & Häyry, M. (2022). The role of philosophers in bioethics. *The American Journal of Bioethics. 22*(12), 58–60.

